# Lysophosphatidic Acid Is Associated With Cardiac Dysfunction and Hypertrophy by Suppressing Autophagy via the LPA3/AKT/mTOR Pathway

**DOI:** 10.3389/fphys.2018.01315

**Published:** 2018-09-18

**Authors:** Jinjing Yang, Jiyao Xu, Xuebin Han, Hao Wang, Yuean Zhang, Jin Dong, Yongzhi Deng, Jingping Wang

**Affiliations:** ^1^Department of Cardiology, Shanxi Cardiovascular Disease Hospital, Taiyuan, China; ^2^Shanxi Cardiovascular Disease Institute, Taiyuan, China; ^3^Central Laboratory, Shanxi Cardiovascular Disease Hospital, Taiyuan, China; ^4^The Affiliated Cardiovascular Disease Hospital of Shanxi Medical University, Taiyuan, China; ^5^Department of Cardiovascular Surgery, Shanxi Cardiovascular Disease Hospital, Taiyuan, China

**Keywords:** lysophosphatidic acid, cardiomyocyte hypertrophy, autophagy, myocardial infarction, mammalian target of rapamycin (mTOR)

## Abstract

**Background:** Lysophosphatidic acid (LPA), as a phospholipid signal molecule, participates in the regulation of various biological functions. Our previous study demonstrated that LPA induces cardiomyocyte hypertrophy *in vitro*; however, the functional role of LPA in the post-infarct heart remains unknown. Growing evidence has demonstrated that autophagy is involved in regulation of cardiac hypertrophy. The aim of the current work was to investigate the effects of LPA on cardiac function and hypertrophy during myocardial infarction (MI) and determine the regulatory role of autophagy in LPA-induced cardiomyocyte hypertrophy.

**Methods:** *In vivo* experiments were conducted in Sprague-Dawley rats subjected to MI surgery or a sham operation, and rats with MI were assigned to receive an intraperitoneal injection of LPA (1 mg/kg) or vehicle for 5 weeks. The *in vitro* experiments were conducted in H9C2 cardiomyoblasts.

**Results:** LPA treatment aggravated cardiac dysfunction, increased cardiac hypertrophy, and reduced autophagy after MI *in vivo*. LPA suppressed autophagy activation, as indicated by a decreased LC3II-to-LC3I ratio, increased p62 expression, and reduced autophagosome formation *in vitro*. Rapamycin, an autophagy enhancer, attenuated LPA-induced autophagy inhibition and H9C2 cardiomyoblast hypertrophy, while autophagy inhibition with Beclin1 siRNA did not further enhance the hypertrophic response in LPA-treated cardiomyocytes. Moreover, we demonstrated that LPA suppressed autophagy through the AKT/mTOR signaling pathway because mTOR and PI3K inhibitors significantly prevented LPA-induced mTOR phosphorylation and autophagy inhibition. In addition, we found that knockdown of LPA3 alleviated LPA-mediated autophagy suppression in H9C2 cardiomyoblasts, suggesting that LPA suppresses autophagy through activation of the LPA3 and AKT/mTOR pathways.

**Conclusion:** These findings suggest that LPA plays an important role in mediating cardiac dysfunction and hypertrophy after a MI, and that LPA suppresses autophagy through activation of the LPA3 and AKT/mTOR pathways to induce cardiomyocyte hypertrophy.

## Introduction

Cardiac hypertrophy is considered to be a crucial risk factor for heart failure and eventually leads to systolic dysfunction, although cardiac hypertrophy is a fundamental feature of cardiac adaptation to diverse neurohumoral stimuli secondary to myocardial infarction (MI) during the early stages (Mathew et al., 2001). Cardiac hypertrophy is finely regulated by a number of molecular signaling pathways.

Lysophosphatidic acid (LPA) is mainly generated by autotaxin (ATX) and is the simplest glycerophospholipid (Moolenaar and Perrakis, 2011). As an extracellular bioactive phospholipid, LPA is widely involved in the regulation of various cellular processes, such as apoptosis, proliferation, differentiation, and migration (Mills and Moolenaar, 2003; Ye et al., 2005; Zhang et al., 2007; Yu et al., 2012; Taniguchi et al., 2017), via activation of at least six known G protein-coupled receptors (GPCRs [LPA1–LPA6]) (Hecht et al., 1996; Contos and Chun, 2000, 2001; Yung et al., 2014; Taniguchi et al., 2017). It has been reported that serum LPA levels are significantly elevated in patients with acute myocardial infarction (MI) (Chen et al., 2003). We previously reported that LPA induces cardiomyocyte hypertrophy *in vitro* (Yang et al., 2013). Moreover, LPA1 and LPA3 expression are increased in rat hearts after MI (Chen et al., 2008), implying that LPA might be involved in cardiac remodeling after a MI. The effects of LPA on cardiac function and myocardial hypertrophy during MI; however, have not been elucidated.

Autophagy is an evolutionarily conservative and physiologic process and serves to maintain normal cell function and structure by catabolizing cytoplasmic proteins and damaged organelles via lysosomes (DeMartino, 2018). Dysregulation of autophagy has been suggested to be associated with multiple disease conditions, including cardiac hypertrophy, dilated cardiomyopathy, and heart failure (Tanaka et al., 2000; Nakai et al., 2007). A number of studies have suggested that autophagy, which is increased in the post-infarction heart (Kanamori et al., 2011), protects the heart against cardiac remodeling after a MI (Buss et al., 2009; Wu et al., 2014), although overactivation of autophagy might be harmful to the heart subjected to ischemia/reperfusion injury (Matsui et al., 2007; Chen-Scarabelli et al., 2014). Regulation of myocyte autophagy in cardiac remodeling, such as myocyte hypertrophy, is not fully understood. It has been reported that LPA inhibits autophagy in starvation-induced cancer cells (Chang et al., 2007) and in injured carotid artery tissues (Shen et al., 2018). Moreover, LPA is capable of regulating activation of the mammalian target of rapamycin (mTOR) pathway (Kam and Exton, 2004; Lee et al., 2016), which negatively mediates autophagy in various cells. Thus, we hypothesized that LPA suppresses autophagy to induce cardiomyocyte hypertrophy.

The present study investigated the effects of LPA on cardiac function and hypertrophy during MI and determined whether or not autophagy is involved in LPA-mediated cardiomyocyte hypertrophy.

## Materials and Methods

The study was approved by the Animal Care Committee of Shanxi Medical University. The animal experiments conformed to the Guide for the Care and Use of Laboratory Animals according to the Beijing Ethical Review Council on Animal Care (1996).

### Materials

Lysophosphatidic acid (LPA) (oleoyl C: 18:1) was obtained from Avanti Polar Lipids (Alabaster, AL, United States). 3-(4-[4-([1-(2-chlorophenyl)ethoxy]carbonylamino)-3-methyl-5-isoxazolyl]benzylsulfanyl)propanoic acid (Kil6425) was purchased from Sigma (St. Louis, MO, United States). PI3K inhibitor LY294002, rapamycin, anti-phosphorylated mTOR (ser2448) antibody, anti-total mTOR antibody, anti-p70s6 kinase (Thr389) antibody, anti-phosphorylated p70s6 kinase, anti-4E-BP1 antibody, anti-phosphorylated 4E-BP1(Thr37/46) antibody, and anti-phosphorylated AMPKα (Thr172) antibody were obtained from Cell Signaling Technology (Beverly, MA, United States). Anti-p62/SQSTM1 (sequestosome1) polyclonal antibody and anti-beclin1 polyclonal antibody were from Proteintech Technology (Proteintech Group, Wuhan, China), and anti-LC3B polyclonal antibody and anti-GAPDH monoclonal antibody were obtained from Sigma (St. Louis, MO, United States). Lipofectamine^TM^ RNAiMAX, stealth siRNA, and siRNA negative control were purchased from life Technologies (Invitrogen, Carlsbad, CA, United States). The PowerUp^TM^ SYBR Green Master Mix assay (Applied Biosystems, Life Technologies, Foster City, CA, United States).

### Animals

In the present study, male Sprague-Dawley rats weighing 200 g were obtained from the Shanxi Medical University Animal Centre. All animals were housed in standard cages in a temperature-controlled (22–25°C) room on a 12-h light and 12-h dark cycle. The rats were fed with standard chow and allowed to drink water freely.

The MI rat model was established as described previously (Fan et al., 2015). In brief, 1% pentobarbital sodium (40 mg/kg body weight) was intraperitoneally injected as an anesthetic agent. The rats were ventilated with a rodent respirator and underwent thoracotomies between the fourth and fifth intercostal space. The left anterior descending coronary (LAD) artery was ligated with a 7-0 size polypropylene suture to construct the MI rat model, and occlusion was confirmed by blanching of the left ventricle anterior wall. The animals were randomly divided into the following three groups (*n* = 6–7 per group): sham operation; MI + PBS; and MI + LPA treatment. For the sham operation, rats were subjected to the same surgical procedure; however, the ligature around the LAD artery was not tied. For drug administration, rats with MI were assigned to receive intraperitoneal injections of LPA (1 mg/kg per day) or vehicle (0.1% fatty acid-free bovine serum albumin/PBS solution) 3 days after the MI. Five weeks after surgery, animal cardiac function and structure were tested by small animal echocardiography, then the animals were sacrificed by intravenous injection of 10% KCL, and the hearts were arrested in diastole and removed for further biochemical and histologic analyses.

### Echocardiographic Analysis for Cardiac Structure and Function

Echocardiographic was used to assess cardiac structure and function in anesthetized rats. M-mode images were obtained to determine left ventricular dimensions, including the left ventricular internal diameter at end diastole (LVIDd), left ventricular internal diameter at end systole (LVIDs), left ventricular ejection fraction (LVEF), left ventricular posterior wall thickness at end diastole (LVPWd), left ventricular posterior wall thickness at end systole (LVPWs) and LVEF, interventricular septum at end diastole (IVSd), interventricular septum at end systole (IVSs). Fractional shortening (FS) was calculated as (LVIDd-LVIDs)/LVIDd × 100. The pooled data were analyzed for statistical significance.

### Culture and Treatment of H9C2 Cardiomyoblasts

H9C2 cardiomyoblasts were obtained from the Cell Bank at Shanghai Institute for Biological Sciences and cultured with DMEM (Gibco, CA, United States) containing 10% fetal bovine serum (Gibco, CA, United States), D-glucose (4.5 g/L) and penicillin/streptomycin (1000 U/ml of each) at a density of 0.1 × 10^6^ cells/35 mm in 6-well plates. The cells were then incubated at 37°C in a humidified atmosphere with 5% CO_2_ and 95% air. When H9C2 cardiomyoblasts were grown to 80% confluence, the culture medium was replaced with serum-free DMEM for 24 h. The cardiomyocytes were used for experiments after overnight serum starvation. When H9C2 cardiomyoblasts were grown to 30–50% confluence, they were used for the Fluorescent Staining experiments.

### Quantitative Reverse Transcription-Polymerase Chain Reaction (qRT-PCR)

Total RNA was extracted from cultured H9C2 cardiomyoblasts with Trizol (Invitrogen, Carlsbad, CA, United States), then quantified on a spectrophotometer. cDNA was generated from total RNA (1 μg) using reverse transcriptase and oligo (dT) 15 primer. The PowerUp^TM^ SYBR Green Master Mix assay (Applied Biosystems, Life Technologies, Foster City, CA, United States) was used to detect and quantify the mRNA levels of each gene in an Applied Biosystems 7300 (Foster City, CA, United States).

All specific sequence primers included in the present work were listed as follows: atrial natriuretic peptide (ANP): 5′-GGG TAG GAT TGA CAA GGA TTG G-3′ and 5′-CTC CAG GAG GGT ATT CAC CAC-3′; brain natriuretic peptide (BNP):5′-CTC CAG AAC AAT CCA CGA TGC-3′ and 5′-CTT CCT AAA ACA ACC TCA GCC-3′; LPA3: 5′-TGT CAA CCG CTG GCT TCT-3′ and 5′-CAG TCA TCA CCG TCT CAT TAG-3′; Beclin1: 5′-TAC TGT TCT GGG GGT TTG CG5′- and 5′-GAA CTT GAG CGC CTT TGT CC-3′; GAPDH: 5′-CAA CGA CCC CTT CAT TGA CCT-3′ and 5′-CAG TAG ACT CCA CGA CAT ACT C-3′; beta-actin: 5′-GAA CCC TAA GGC CAA CCG TGA A-3′ and 5′-TAC GTA CAT GGC TGG GGT GT -3′. In this work, GAPDH or beta-actin were used as reference genes for data normalization. The relative expression of the target gene was normalized to the reference gene using the comparative 2^-ΔΔ^*^C^*^t^ method.

### Fluorescent Staining and Cell Surface Area of H9C2 Cardiomyoblasts

H9C2 cardiomyoblasts were cultured on cover slides, placed in 6-well culture plates, and treated with LPA (10 μM) alone for 24 h or pre-treated with rapamycin (a mTOR inhibitor) for 1 h before LPA treatment. H9C2 cardiomyoblasts were stained with TRITC-conjugated phalloidin solution from Sigma (St. Louis, MO, United States) for measurement of the cell surface area as previously described (Yang et al., 2013). In brief, cells were fixed with 4% paraformaldehyde and permeabilized with 0.1% Triton X-100 for 10 min at room temperature. After washing, cells were stained with TRITC-conjugated phalloidin at a concentration of 1 μg/ml for 1 h at 37°C. Then, DAPI staining was performed to mark the nuclei. The stained cells were imaged with a laser confocal microscope (TCS NT; Leica, Wetzlar, Germany) and assessed with ImageJ software. At least 50 H9C2 cardiomyoblasts were examined in each group.

### Transfection of siRNA Into H9C2 Cardiomyoblasts

In the transfection experiments, H9C2 cardiomyoblasts were transfected with siRNA of Beclin1or LPA3 for 24 h, then stimulated with LPA for 4 h or 24 h. H9C2 cardiomyoblasts were transfected with small interfering RNA (siRNA) for Beclin1 or LPA3 using Lipofectamine^TM^ RNAiMAX (Invitrogen, Carlsbad, CA, United States), as previously described (Yang et al., 2013). The Beclin1 Stealth siRNA duplex target sequences were 5′-UACACCACCACCAUGAUGAAGAAGG-3′ and 5′-CCU UCU UCA UCA UGG UGG UGG UGU A-3′. The LPA3 Stealth siRNA duplex target sequences were 5′-UAC ACC ACC ACC AUG AUG AAG AAG G-3′ and 5′-CCU UCU UCA UCA UGG UGG UGG UGU A-3′. The sequence for the stealth siRNA low-GC duplex was used as a negative control. The cells were transfected with the stealth siRNA at 30 nM.

### Western Blot Analysis

Western blots were performed according to standard procedures, as previously described (Yang et al., 2013). H9C2 cardiomyoblasts or rat LV myocardial tissue samples were lysed and homogenized in a RIPA lysis buffer containing 50 mM Tris (pH = 7.4), 150 mM NaCl, 1% Triton X-100, 1 mM EDTA 1% sodium deoxycholate, 1 mM dithiothreitol (DTT), 20 mM HEPES (pH = 7.5), 1 mM β-glycerolphosphate, 1 mM phenylmethyl sulfonylfluoride [PMSF] (Boster Biological Technology, Wuhan, China), and 10 μg/ml each of leupeptin, aprotinin, and pepstatin. The extract protein concentration was quantified with the BCA assay (Beyotime Biotechnology, Beijing, China). Equal amounts of protein (30–40 μg/lane) were subjected to 12% or 8% SDS-PAGE electrophoresis and transferred to nitrocellulose membranes. The membranes were blocked in 5% skim milk for 2 h at room temperature, then the blots were incubated overnight with primary antibodies at 4°C. After washing, the blots were incubated with goat anti-rabbit IgG peroxidase-conjugated secondary antibodies for 1 h at 37°C. Western blot bands were displayed by chemiluminescence and quantified using Image LabTM analysis software. Protein bands were normalized to the control sample.

The primary antibodies were purchased from Cell Signaling Technology (Beverly, MA, United States), including anti-phosphorylated mTOR (ser2448) antibody (1:1000), anti-total mTOR antibody (1:1000), anti-p70s6 kinase (Thr389) antibody (1:1000), anti-phosphorylated p70s6 kinase (1:1000), anti-4E-BP1 antibody (1:1000), anti-phosphorylated 4E-BP1 (Thr37/46) antibody (1:1000), and anti-phosphorylated AMPKα (Thr172) antibody (1:1000). Anti-p62/SQSTM1 (sequestosome1) polyclonal antibody (1:1000) and anti-beclin1 polyclonal antibody (1:1000) were from Proteintech Technology (Proteintech Group, Wuhan, China), and anti-LC3B polyclonal antibody (1:1000) and anti-GAPDH monoclonal antibody (1:5000) were obtained from Sigma (St. Louis, MO, United States).

### Hematoxylin and Eosin Staining

Hematoxylin and eosin staining for cardiac tissues was carried out to demonstrate the cross-sectional area of cardiac myocytes, and tissue images were examined under a light microscope. Myocyte size was determined by measurement of myocyte cross-sectional area and quantified using Image Pro Plus software. Fifty myocytes per group were measured for the data analysis.

### Enzyme Linked Immunosorbent Assay (Elisa)

Levels of troponin I in plasma from rats were quantified by the rat troponin I ELISA Kit (Elabscience Biotechnology, Wuhan, China) according to the manufacturer’s instructions. Sensitivity: 9.38 pg/ml. Detection Range: 15.63–1000 pg/ml. Plasma LPA concentrations were quantified using competitive enzyme-linked immunosorbent assay (ELISA) Kit for LPA (Cloud-Clone Corp., Houston, TX, United States) following the manufacturer’s instructions. Detection Range: 123.5–10000 ng/ml.

### Transmission Electron Microscopy for Analysis of Autophagosomes

Transmission electron microscopy analysis was performed to detect autophagosomes in H9C2 cardiomyoblasts. Briefly, cells were collected from the cultured plates and fixed using 2.5% glutaraldehyde solution at 4°C for 2 h. Then, cells were washed and post-fixed with 1% osmic acid at 4°C for 1 h. After dehydration in methanol in a serial concentration gradient, cells were embedded in Embed-812 medium. An Ultrotome (Reichert Ultracuts; Leica) was used to make ultrathin sections of cells on uncoated copper grids. The ultrathin sections were stained with 0.2% lead citrate/1% uranyl acetate. Cell images were captured under a transmission electron microscope (JEM1230; JEOL, Japan).

### Cell Counting Kit-8 Assay

H9C2 cardiomyoblasts were seeded in 96-well plates at a concentration of 10000 cells per well, and treated with 10 μM LPA for various lengths of time (6–48 h). Cell proliferation was assessed using Cell Counting Kit-8(CCK-8) assay (DOJINDO, Kumamoto, Kyushu, Japan).

### Statistical Analysis

Data were presented as the mean ± standard error of the mean (SEM). Differences among the groups were tested by one-way ANOVA followed by a Tukey’s *post hoc* test. When there were only two means to compare, a Student’s *t*-test was used. A *P* < 0.05 was considered statistically significant. All data were analyzed using SPSS version 16.0.

## Results

### LPA Promotes Cardiac Dysfunction and Cardiac Hypertrophy *in vivo*

**FIGURE 1:**
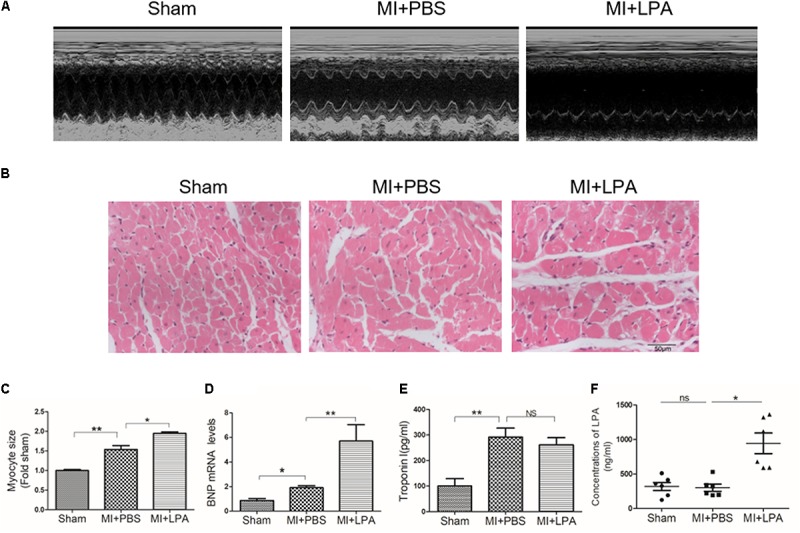
Lysophosphatidic acid (LPA) aggravates cardiac dysfunction and hypertrophy *in vivo*. **(A)** Representative M-mode echocardiography images of hearts in each group. **(B)** Representative hematoxylin and eosin staining of cardiac tissue section in each group. Scale bars, 50 μm. **(C)** The analysis of myocyte size. *n* = 4 rat per group, ^∗^*P* < 0.05, ^∗∗^*P* < 0.01. **(D)** The analysis of BNP mRNA levels, *n* = 4 rat per group, ^∗^*P* < 0.05, ^∗∗^*P* < 0.01. **(E)** The Elisa assay was used to determine the troponin I concentrations in plasma, *n* = 4 rat per group, ^∗∗^*P* < 0.01, ^NS^*p* > 0.05. **(F)** The Elisa assay of the LPA concentrations in plasma 5 weeks after MI, *n* = 6 rat per group, ^∗∗∗^*P* < 0.001, ^NS^*p* > 0.05. All data were presented as mean ± SEM. One-way ANOVA followed by a Tukey’s *post hoc* was used for multiple testing.

Our previous study showed that LPA induces cardiomyocyte hypertrophy *in vitro* (Yang et al., 2013). To investigate the effects of LPA on cardiac function and hypertrophy induced by MI challenge, rats underwent the MI operation and were treated with LPA or PBS for 5 weeks. As presented in **Table 1**, compared with sham-operated rats, MI rats displayed an apparent cardiac dysfunction. Importantly, LPA treatment (1 mg/kg per day) further decreased the EF and FS in rat hearts after MI, indicating that LPA aggravated cardiac dysfunction in the post-infarct heart (**Figure 1A**). Interestingly, we observed that LPA treatment (1 mg/kg per day) promoted cardiac hypertrophy, as indicated by hematoxylin and eosin staining of cardiac tissues, measurement of myocyte size, and natriuretic peptide B (BNP) expression (**Figures 1B–D**). In addition, we also found that the plasma levels of troponin I were increased in MI rats; however, there were no significant differences in troponin I levels between the LPA-treated and MI-operated groups (**Figure 1E**). Moreover, LPA infusion (1 mg/kg per day) caused an increase in the plasma concentrations of LPA compared with the MI-treated groups at 5 weeks post-MI (**Figure 1F**). There results demonstrate that LPA promotes cardiac dysfunction and hypertrophy following MI *in vivo*.

**Table 1:** Echocardiographic parameters in each group 5 weeks after MI.

	Sham (*n* = 6)	MI + PBS (*n* = 6)	MI + LPA (*n* = 7)
LVEF (%)	88 ± 3.25	52.33 ± 2.73^∗∗∗^	40.85 ± 2.35^∗∗∗#^
FS (%)	53.50 ± 4.07	23.67 ± 1.45^∗∗∗^	17.43 ± 1.15^∗∗∗#^
LVIDd (mm)	5.14 ± 0.59	6.63 ± 0.77	7.19 ± 0.70
LVIDs (mm)	2.65 ± 0.45	5.11 ± 0.63^∗^	5.95 ± 0.58^∗∗^
LVPWd (mm)	1.59 ± 0.05	2.09 ± 0.15	1.54 ± 0.27
LVPWs (mm)	2.82 ± 0.19	2.73 ± 0.19	2.19 ± 0.30
IVSd (mm)	1.43 ± 0.22	1.16 ± 0.17	1.03 ± 0.17
IVSs (mm)	2.62 ± 0.22	1.61 ± 0.18^∗∗^	1.37 ± 0.20^∗∗^
HW/BW (mg/g)	2.91 ± 0.09	3.37 ± 0.09^∗^	3.84 ± 0.13^∗∗∗#^
HR (beat/min)	359 ± 28	373 ± 51	383 ± 47


*LVEF, left ventricular ejection fraction; FS, fractional shortening; LVIDd, left ventricular internal diameter at end diastole; LVIDs, left ventricular internal diameter at end systole; LVPWd, left ventricular posterior wall thickness at end diastole; LVPWs, left ventricular posterior wall thickness at end systole; IVSd, interventricular septum at end diastole, IVSs interventricular septum at end systole; HW/BW: heart weight/body weight; HR: heart rate. ^∗^p < 0.05 vs. sham group, ^∗∗^p < 0.01 vs. sham group, ^∗∗∗^p < 0.001 vs. sham group. ^#^p < 0.05 vs. MI + PBS group. Values were presented as mean ± SEM. One-way ANOVA followed by a Tukey’s post hoc was used for multiple testing*.

### LPA Down-Regulates Autophagy Activity *in vitro* and *in vivo*

**FIGURE 2:**
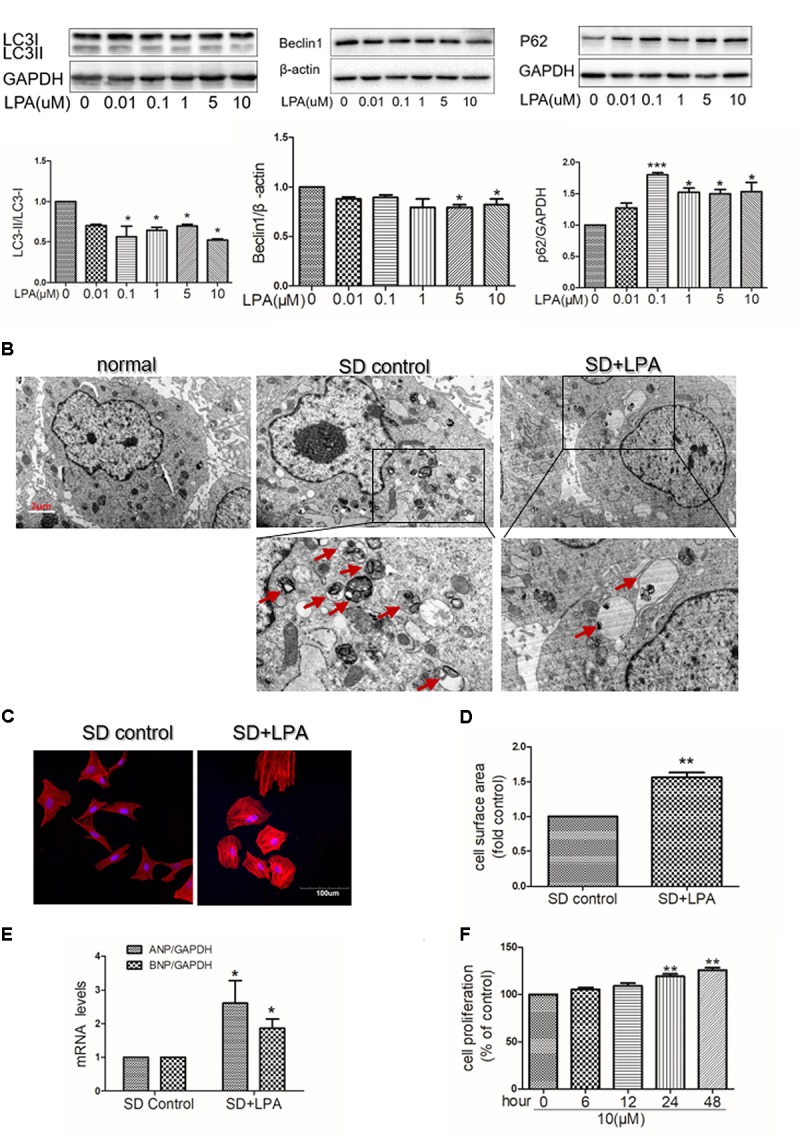
Lysophosphatidic acid down-regulates autophagy activity *in vitro*. **(A)** Serum-starved (SD) H9C2 cardiomyoblasts were treated with different concentrations of LPA for 4 h. And then the protein levels of LC3B, Beclin1 and P62 were assessed by Western blotting. ^∗^*P* < 0.05, ^∗∗∗^*P* < 0.05 vs. control. **(B)** Serum-starved H9C2 cardiomyoblasts were treated with 10 μM LPA for 4 h. Representative electron micrographs demonstrating decreased autophagic vacuole formation (red arrows) in the LPA-treated group compared with the control group. Scar bar: 2 μm. **(C)** Serum-starved H9C2 cardiomyoblasts were treated with 10 μM LPA stimulation for 24 h. Sarcomere organization was stained with TRITC-conjugated phalloidin for F-actin (red). DAPI staining marked the nuclei. Scale bars, 100 μm. **(D,E)** The analysis of cell surface area and mRNA levels of ANP and BNP. *n* = 3, ^∗^*P* < 0.05, ^∗∗^*P* < 0.01 versus SD control. **(F)** H9C2 cardiomyoblasts were treated with 10 μM LPA for various lengths of time (6–48 h), cell proliferation was assessed by CCK-8 assay *n* = 3, ^∗∗^*P* < 0.01 vs. control. All data represents mean ± SEM. Each experiment was performed thrice and shown results were representative. A one-way ANOVA followed by a *post hoc* Tukey’s test was used for multiple testing. Student’s t-tests were used to compare two groups of data.

**FIGURE 3:**
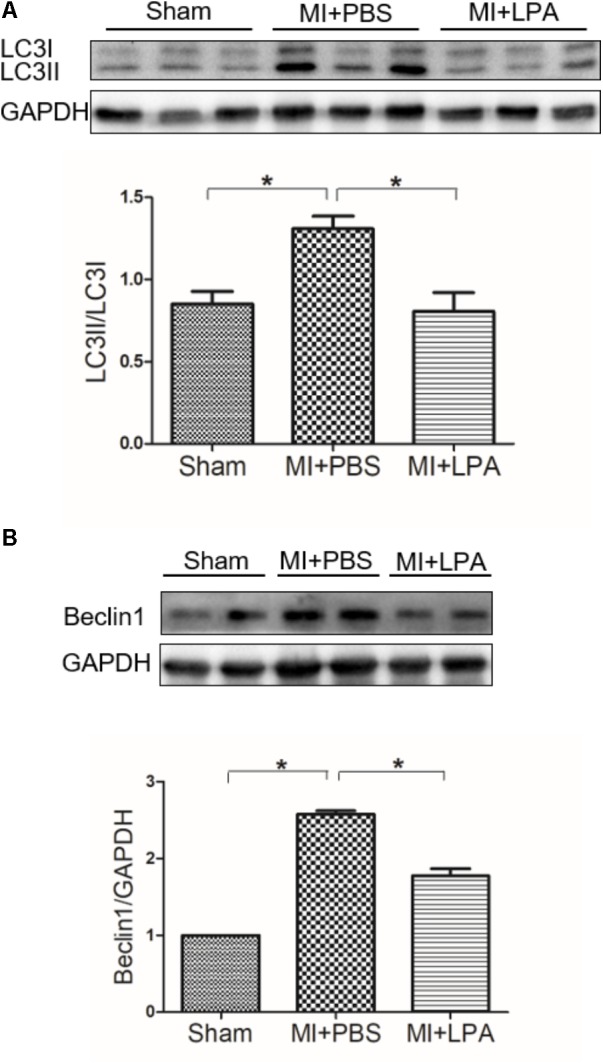
Lysophosphatidic acid down-regulates autophagy activity *in vivo*. **(A,B)** Western blotting analysis of the protein levels of LC3B and Beclin1 in each group. *n* = 4 rat per group, values represents mean ± SEM. ^∗^*P* < 0.05. Shown results were representative. A one-way ANOVA followed by a *post hoc* Tukey’s test was used for multiple comparison.

To determine whether or not autophagy plays a functional role in LPA-induced cardiomyocyte hypertrophy, we first explored the effect of LPA on autophagic activity. The marker protein of autophagy was determined by Western blotting in H9C2 cardiomyoblasts treated with different concentrations of LPA (0.01, 0.1, 1, 5, and 10 μM) for 4 h. As shown in **Figure 2A**, LPA caused a concomitant decrease in the LC3 II-to-I ratio and Beclin1 expression with a significant role at the 10 μM LPA dose. Moreover, LPA significantly increased P62/SQSTM1 expression in H9C2 cardiomyoblasts. Representative electron micrographs demonstrated decreased autophagic vacuole formation in the LPA-treated group compared with the serum-starved (SD) control group when H9C2 cardiomyoblasts were treated by 10 μM LPA for 4 h, indicating that LPA down-regulated autophagy in H9C2 cardiomyoblasts (**Figure 2B**). LPA also induced a hypertrophic response in H9C2 cardiomyoblasts, as assessed by sarcomere organization staining for sarcomeric F-actin, analysis of the cell surface area, and assessment of hypertrophic markers [ANP and BNP] (**Figures 2C–E**). CCK-8 assay showed that LPA promoted H9C2 cardiomyoblasts proliferation (**Figure 2F**). In addition, consistent with the *in vitro* experiment, LPA treatment caused down-regulation of autophagy in cardiac tissues, as shown by the decreased LC3II-to-LC3I ratio and Beclin1 expression (**Figures 3A,B**). These findings indicate that LPA inhibits autophagy *in vivo* and *in vitro*.

### Autophagy Is Involved in LPA-Induced Hypertrophy in H9C2 Cardiomyoblasts

**FIGURE 4:**
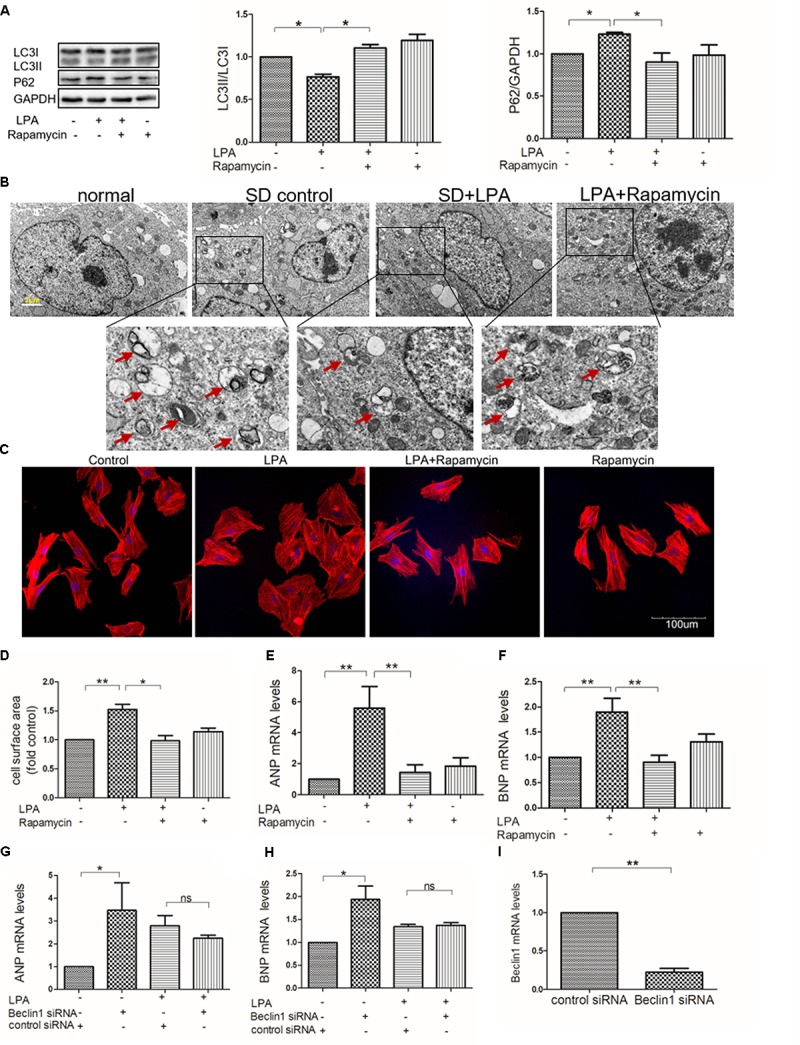
Autophagy is involved in LPA-induced hypertrophy in H9C2 cardiomyoblasts. **(A)** H9C2 cardiomyoblasts were treated with 50 nM rapamycin (an autophagy enhancer) for 1 h, before 10 μM LPA stimulation for 4 h. The protein levels of LC3B and p62 were detected by Western Blotting. *n* = 3, ^∗^*P* < 0.05. **(B)** Representative electron micrographs of autophagic vacuole formation in H9C2 cardiomyoblasts (red arrows). Scale bars, 2 μm. **(C)** H9C2 cardiomyoblasts were treated with 50 nM rapamycin for 1 h, before 10 μM LPA stimulation for 24 h. Sarcomere organization determined by TRITC-conjugated phalloidin for F-actin (red). DAPI staining marked the nuclei. Scale bars, 100 μm. The assessment of cell surface area and mRNA levels of ANP and BNP **(D–F)**. *n* = 3, ^∗^*P* < 0.05, ^∗∗^*P* < 0.01. H9C2 cardiomyoblasts were transfected with siRNA of Beclin1 for 24 h, and then exposed to 10 μM LPA for 24 h. The mRNA levels of ANP and BNP **(G,H)**. *n* = 3, ^∗^*P* < 0.05, ^ns^*p* > 0.05. H9C2 cardiomyoblasts were transfected with siRNA of Beclin1 for 24 h. Then the mRNA levels of Beclin1 were analyzed by qRT-PCR **(I)**. *n* = 3, ^∗∗^*P* < 0.01. All data represents mean ± SEM. Each experiment was performed thrice and shown results were representative ones. A one-way ANOVA followed by a *post hoc* Tukey’s test was used for multiple comparison. Student’s *t*-tests were used to compare two groups of data.

Several studies have shown that autophagy is involved in regulation of cardiac hypertrophy induced by various stresses, such as ischemia (Delbridge et al., 2017) and oxidative stress (Dai et al., 2011). To determine whether or not autophagy is involved in LPA-induced cardiomyocyte hypertrophy, an autophagy activator (rapamycin) was used. We found that rapamycin increased the LC3 II-to-I ratio, decreased P62/SQSTM1 expression, and increased autophagic vacuole formation in H9C2 cardiomyoblasts incubated with LPA (**Figures 4A,B**). Moreover, rapamycin attenuated LPA-induced H9C2 cardiomyoblasts hypertrophy, as demonstrated by visualization of sarcomere organization, measurement of the cell surface area, and assessment of hypertrophic indicators (ANP and BNP) (**Figures 4C–F**). To further confirm the role of autophagy in LPA-induced cardiomyocyte hypertrophy, the autophagic protein, Beclin1, was knocked down through the transfection of Beclin1 siRNA. Knockdown of Beclin1 had no additional effect on the LPA-induced hypertrophic response in H9C2 cardiomyoblasts (**Figures 4G–I**). These results demonstrate that autophagy might be involved in LPA-induced H9C2 cardiomyoblast hypertrophy.

### AKT/mTOR Pathway Is Required for LPA-Induced Autophagy Inhibition

**FIGURE 5:**
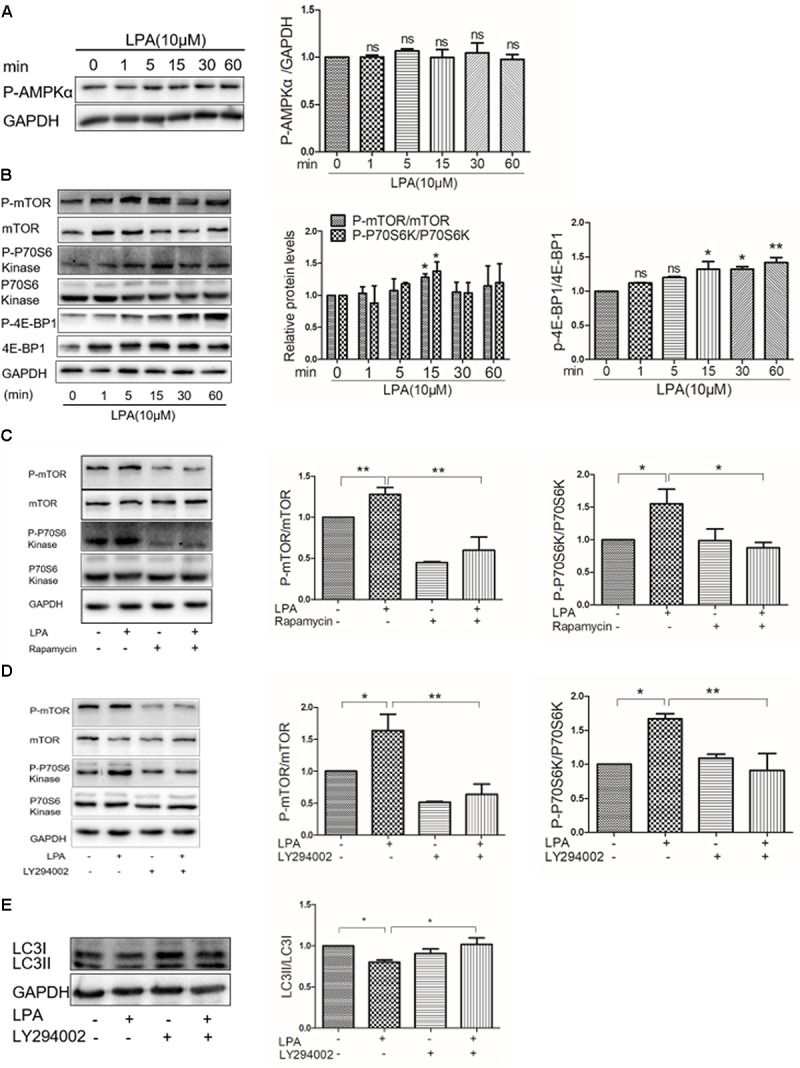
Lysophosphatidic acid down-regulates autophagy through the AKT/mTOR pathway. **(A,B)** Western blotting analysis of the phosphorylation of AMPKα (Thr172), mTOR (Ser2448), P70S6 kinase (Thr389), and 4E-BP1 (Thr37/46) in H9C2 cardiomyoblasts treated with 10 μM LPA for the indicated time. ^∗^*P* < 0.05 vs. control, ^ns^*P* > 0.05. **(C)** H9C2 cardiomyoblasts were treated with 50 nM rapamycin for 1 h, before 10 μM LPA stimulation for 15 min. Then the phosphorylation of mTOR and P70S6 kinase were analyzed by Western blotting. ^∗^*P* < 0.05, ^∗∗^*P* < 0.01. **(D)** H9C2 cardiomyoblasts were treated with 6.25 μM LY294002 for 1 h, and then exposed to 10 μM LPA for 15 min. The phosphorylation of mTOR and p70S6 kinase were analyzed by Western blotting. ^∗^*P* < 0.05, ^∗∗^*P* < 0.01. **(E)** H9C2 cardiomyoblasts were treated with 6.25 μM LY294002 for 1 h, before exposure to 10 μM LPA for 4 h. Then the protein levels of LC3B were determined by Western blotting. ^∗^*P* < 0.05. All data represents mean ± SEM. Each experiment was performed thrice and shown results were representative. A one-way ANOVA followed by a *post hoc* Tukey’s test was used for multiple comparison.

Abundant evidence has shown that the AMP activated protein kinase (AMPK)/mTOR signaling pathway is essential in mediating autophagy (Matsui et al., 2007; Buss et al., 2009, 2010; Herrero-Martin et al., 2009). To determine whether or not AMPK/mTOR signaling is involved in LPA-regulated autophagy in cardiomyocytes, Western blotting analysis was performed to assess phosphorylated AMPK and mTOR in H9C2 cardiomyoblasts exposed to 10 μM LPA at the indicated times. There was no significant decrease in the phosphorylation of AMPKa determined by Western blotting in H9C2 cardiomyoblasts after administration of LPA (**Figure 5A**). Importantly, we found that LPA induced an increase in the phosphorylation of mTOR and its downstream targets (p70S6K and 4E-BP1) (**Figure 5B**), and these effects were abrogated by rapamycin, a mTOR inhibitor (**Figure 5C**). In addition, AKT is an upstream regulatory molecule of the mTOR signaling pathway. Our previous study identified that the PI3K inhibitor, LY294002, attenuates LPA-induced cardiomyocyte hypertrophy *in vitro* (Yang et al., 2013). In this work, we observed that the PI3K inhibitor, LY294002, prevented the LPA–mediated effects on the mTOR pathway and autophagy (**Figures 5D,E**). Together, these data indicate that the AKT/mTOR pathway is involved in mediating LPA-induced autophagy inhibition in H9C2 cardiomyoblasts.

### LPA Down-Regulates Autophagy Through LPA3

**FIGURE 6:**
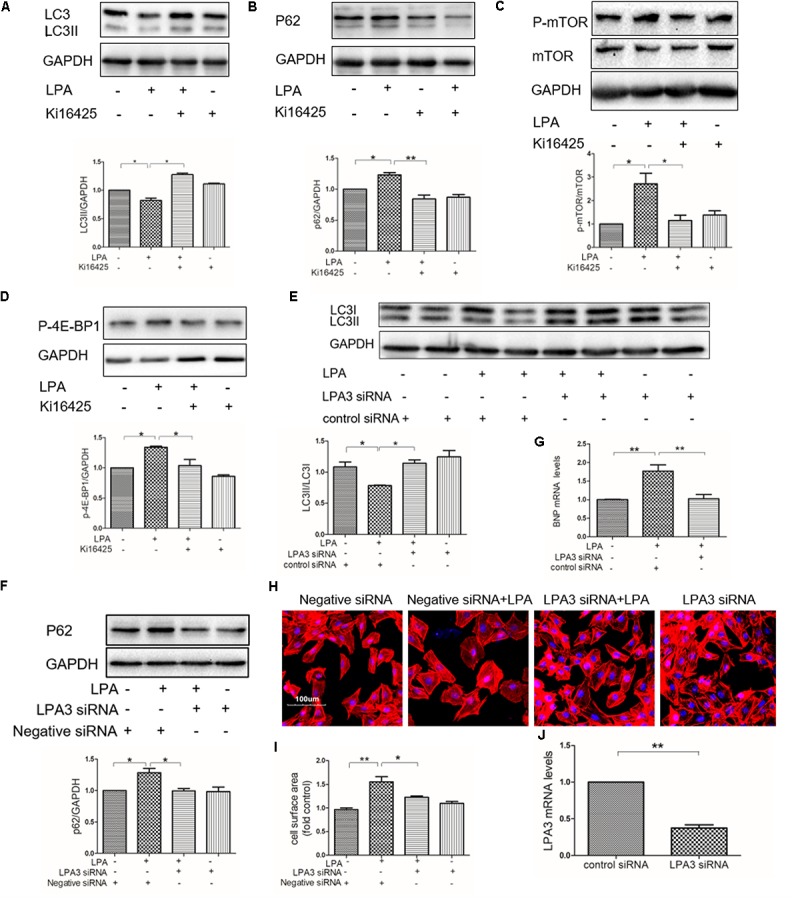
Knockdown of LPA3 attenuates LPA-mediated autophagy inhibition. **(A,B)** Serum-starved H9C2 cardiomyoblasts were treated with 10 μM Ki16425 for 1 h, before exposure to 10 μM LPA for 4 h. Then the protein levels of LC3B and P62 were tested by Western blotting. ^∗^*P* < 0.05, ^∗∗^*P* < 0.01. **(C,D)** The phosphorylation of mTOR and 4E-BP1 analyzed by Western blotting in H9C2 cardiomyoblasts treated with 10 μM Ki16425 for 1 h, before 10 μM LPA stimulation for 15 min. ^∗^*P* < 0.05. **(E,F)** H9C2 cardiomyoblasts were transfected with siRNA of LPA3 for 24 h, and then exposed to 10 μM LPA for 4 h. The protein levels of LC3B and p62 were detected by Western blotting. ^∗^*P* < 0.05. **(G)** H9C2 cardiomyoblasts were transfected with siRNA of LPA3 for 24 h, before exposure to 10 μM LPA for 24 h. The mRNA levels of BNP were analyzed by qRT-PCR. *n* = 3, ^∗∗^*P* < 0.01. **(H)** Sarcomere organization determined by TRITC-conjugated phalloidin for F-actin (red). DAPI staining marked the nuclei. Scale bars, 100 μm. **(I)** The assessment of cell surface area. ^∗^*P* < 0.05, ^∗∗^*P* < 0.01. **(J)** Serum-starved H9C2 cardiomyoblasts were transfected with siRNA of LPA3 for 24 h, The mRNA levels of LPA3 were tested by qRT-PCR. *n* = 3, ^∗∗^*P* < 0.01. All data represents mean ± SEM. Each experiment was performed thrice and shown results were representative. A one-way ANOVA followed by a *post hoc* Tukey’s test was used for multiple comparison. Student’s *t*-tests were used to compare two groups of data.

Our previous study showed that LPA induced a hypertrophic response through LPA3 in cardiomyocytes (Yang et al., 2013). To determine whether or not LPA3 is involved in down-regulated autophagy in cardiomyocytes by LPA, the non-selective LPA1/3 antagonist, Ki16425, was used. We found that Ki16425 attenuated the down-regulation of autophagy caused by LPA, as indicated by LC3II and p62 expression (**Figures 6A,B**). Moreover, LPA-induced phosphorylation of mTOR and 4E-BP1 were also alleviated by Ki16425, as shown by the Western blotting of mTOR and 4E-BP1 (**Figures 6C,D**). Moreover, knock-down of LPA3 through siRNA transfection also reduced LPA-mediated effects on autophagy and H9C2 cardiomyoblast hypertrophy (**Figures 6E–I**). Knock-down of LPA3 suppressed LPA3 mRNA levels in H9C2 cardiomyoblasts (**Figure 6J**). These results demonstrate that LPA inhibits autophagy through activation of the LPA3 receptor.

## Discussion

The major findings of this study were as follows: (i) LPA aggravated cardiac dysfunction and promoted cardiac hypertrophy following MI; (ii) LPA down-regulated autophagy *in vivo* and *in vitro*; (iii) autophagy was involved in LPA-induced H9C2 cardiomyoblast hypertrophy; and (iv) LPA down-regulated autophagy through activation of the LPA3 receptor and mTOR signaling pathway. This study revealed the role of exogenous LPA in the heart after a MI. In addition, this data suggested that autophagy is involved in the LPA-induced hypertrophic effect.

LPA, as a phospholipid signaling molecule, which is produced from circulating lysophosphatidylcholine (LPC) by ATX, is involved in multiple cardiovascular diseases (Smyth et al., 2014; Chen et al., 2017; Gu et al., 2017; Nsaibia et al., 2017; Shen et al., 2018). It has been reported that LPA is present in abundance in the serum of patients with an acute myocardial infarction (AMI) (Chen et al., 2003), and LPA1 and LPA3 receptor protein levels are increased in the ischemic heart that develops cardiac remodeling, implying that LPA plays a role in cardiac remodeling after a MI. The current study showed that exogenous LPA worsens systolic function in terms of decreased EF and FS, suggesting that LPA is involved in regulation of cardiac function after a MI. Previous studies have shown that LPA induces cardiomyocyte hypertrophy in primary neonatal rat cardiac myocytes (Hilal-Dandan et al., 2004; Chen et al., 2008; Yang et al., 2013). In the current study, we showed that LPA promotes cardiac hypertrophy *in vivo*, suggesting that LPA might cause cardiac dysfunction by inducing cardiac remodeling. In addition, LPA can mediate diverse actions on cardiac muscle, including modulation of left ventricular systolic and diastolic pressures (Xu et al., 2002), myocardial contractility (Cremers et al., 2003) and electrophysiological instability (Wei et al., 2012). LPA also enhances ventricular arrhythmias in rabbit heart 2 weeks after a MI (Zhang et al., 2016). Therefore, this report did not exclude the possibility that LPA contributes to cardiac dysfunction by other mechanisms, such as regulation of ventricular arrhythmias (Zhang et al., 2016). LPA infusion increased LPA concentrations in plasma at 5 weeks post-MI. It may be associated with the inflammatory response induced by LPA. Because LPA stimulated inflammatory cytokines whereas the production of inflammatory cytokines in damaged tissue serve as a signal for increased ATX expression and LPA production to heal the wound (Brindley, 2004; Benesch et al., 2014). If inflammation is unresolved, inflammatory cytokines produced by LPA may cause further ATX expression which in turn increased LPA formation, thereby leading to more cytokine production in a vicious cycle (Benesch et al., 2014). Thus, exogenous LPA can change circulating LPA levels and strengthen LPA signaling in damaged and inflamed tissue, although LPA has an extremely fast turnover. Interestingly, too little LPA might have similar effects as too much LPA. For instance, effects of LPA on angiogenesis are the same in ATX knockout mice and transgenic mice overexpressing ATX (van Meeteren et al., 2006; Teo et al., 2009;Yukiura et al., 2015).

Lysophosphatidic acid has been reported to protect cardiomyocytes against apoptosis (Karliner et al., 2001), and we recently showed that LPA protects immature cardiomyocytes from I/R injury (Chen et al., 2017); however, no significant difference in troponin I levels between the LPA-treated and MI-operated groups was demonstrated. The discrepancy may be attributed to the different experimental models. Lipid phosphate phosphatase 3 (LPP3), is a major enzyme that dephosphorylates LPA. Cardiac-specific inactivation of LPP3 displays cardiac hypertrophy and myocardial dysfunction in mice, indicating that the LPA/LPP3-signaling play an important role in normal function of cardiomyocytes (Chandra et al., 2018). These data indirectly support the findings in the current study. In addition, LPA3 knockout mice exhibited reduced cardiac hypertrophy compared to wild-type mice post-MI (Cai et al., 2017), indicating that LPA/LPA3 signaling is required for cardiac hypertrophy after MI. In terms of regulation of cardiac hypertrophy, this report supports the mention in the present study.

Accumulating evidence demonstrated that activation of autophagy is increased in ischemic hearts and serves as a protective mechanism against adverse cardiac remodeling in the post-infarction heart (Kanamori et al., 2011; Maeda et al., 2013; Wu et al., 2014). In agreement with previous studies, we also observed that autophagic protein markers were increased in the post-infarction heart. Previous evidence has shown that LPA inhibits autophagy in starvation-induced cancer cells (Chang et al., 2007). Recently, LPA has been reported to promote neointimal hyperplasia after vascular injury by regulating autophagy (Shen et al., 2018). In the present study, the results showed that LPA suppresses activation of autophagy *in vivo* and *in vitro*. Moreover, *in vitro* experiments showed that activation of autophagy through rapamycin not only counteracted autophagy suppression, but also attenuated the induction of H9C2 cardiomyoblast hypertrophy by LPA. These results demonstrated that autophagy suppression is involved in LPA-induced cardiomyocyte hypertrophy.

AMP activated protein kinase, as a central energy sensor that regulates cellular metabolism to maintain energy homeostasis, induces the activity of autophagy (Matsui et al., 2007; Herrero-Martin et al., 2009). Conversely, the AKT/mTOR signaling pathway, which could be activated by LPA (Kam and Exton, 2004; Lee et al., 2016), is thought to be an important negative regulator of autophagy (Zhou et al., 2015; Yang et al., 2018). In the current study, LPA had no significant effect on the phosphorylation of AMPKα; however, LPA promoted the phosphorylation of mTOR and its downstream targets; Furthermore, we indicate that LPA regulated autophagy via the AKT/mTOR pathway in H9C2 cardiomyoblasts because LPA-mediated autophagy inhibition was significantly prevented by mTOR and PI3K inhibitors. It has been reported that mTOR activity prevents the interaction between AMPK and the autophagy-initiating kinase Ulk1 by phosphorylating Ulk1 Ser 757 (Kim et al., 2011), thereby leading to autophagy inhibition. We found that LPA increased phosphorylated Ulk1 Ser 757 (data not shown). Thus, LPA may disturb the interaction between AMPK and Ulk1 through mTOR activation, eventually leading to autophagy suppression. Further studies will be required in the future. In addition, we found that knockdown of LPA3 blocked LPA-regulated autophagy suppression, indicating that LPA3 is involved in LPA-induced autophagy inhibition. Our data provide evidence that LPA might cause autophagy inhibition through LPA3 and the mTOR pathway. There may be some other mediators responsible for the cardiac hypertrophy promotion of LPA. For example, as protein kinase D1 (PKD-1) activator, LPA promotes angiogenesis and microvascular remodeling (Yoshida et al., 2003; Ren et al., 2011; Ren et al., 2016; Dong et al., 2017), which are closely associated with cardiac hypertrophy. LPA stimulates PKD-1 signaling in our model (data not shown) whereas PKD-1 signaling is required for pathological cardiac remodeling (Fielitz et al., 2008). Thus, it would be interesting to know whether LPA-mediated vascular remodeling and PKD-1 signaling is responsible for cardiac hypertrophy.

There were several limitations in this study. First, the regulatory mechanism of autophagy by which LPA caused cardiac dysfunction and remodeling was not thoroughly investigated *in vivo*. Second, we did not employ specific LPA3 receptor antagonist or LPA3 knockout mice to investigate the pinpoint mechanism of how LPA influence hearts following MI *in vivo*, so further *in vivo* experiments remained necessary. In addition, some experiments should be performed in primary cells. Further investigation is required in the future.

In summary, this study demonstrated that LPA contributes to cardiac dysfunction and hypertrophy *in vivo*. *In vitro* experiments suggested that LPA induces H9C2 cardiomyoblast hypertrophy by down-regulating autophagy, which is dependent on the LPA3/AKT/mTOR pathway. These findings provided the first evidence to identify a novel role of LPA in the post-infarction heart and reveal a link between LPA and autophagy in cardiomyocyte hypertrophy. Therefore, targeting LPA signaling might be a therapeutic strategy for the ischemic heart and cardiac hypertrophy.

## Author Contributions

JY and JW designed the research. JY, JX, and HW performed the experiments. JY, JD, and YZ analyzed the data. JY wrote the first manuscript. JW, YD, and XH revised the manuscript.

## Conflict of Interest Statement

The authors declare that the research was conducted in the absence of any commercial or financial relationships that could be construed as a potential conflict of interest.

## References

[B1] Benesch M. G. Ko Y. M. McMullen T. P. Brindley D. N. (2014). Autotaxin in the crosshairs: taking aim at cancer and other inflammatory conditions. *FEBS Lett.* 588 2712–2727. 10.1016/j.febslet.2014.02.009 24560789

[B2] Brindley D. N. (2004). Lipid phosphate phosphatases and related proteins: signaling functions in development, cell division, and cancer. *J. Cell. Biochem.* 92 900–912. 10.1002/jcb.20126 15258914

[B3] Buss S. J. Muenz S. Riffel J. H. Malekar P. Hagenmueller M. Weiss C. S. (2009). Beneficial effects of Mammalian target of rapamycin inhibition on left ventricular remodeling after myocardial infarction. *J. Am. Coll. Cardiol.* 54 2435–2446. 10.1016/j.jacc.2009.08.031 20082935

[B4] Buss S. J. Riffel J. H. Katus H. A. Hardt S. E. (2010). Augmentation of autophagy by mTOR-inhibition in myocardial infarction: when size matters. *Autophagy* 6 304–306. 20104016 10.4161/auto.6.2.11135

[B5] Cai L. Fan G. Wang F. Liu S. Li T. Cong X. (2017). Protective role for LPA3 in cardiac hypertrophy induced by myocardial infarction but not by isoproterenol. *Front. Physiol.* 8:356. 10.3389/fphys.2017.00356 28611684 PMC5447740

[B6] Chandra M. Escalante-Alcalde D. Bhuiyan M. S. Orr A. W. Kevil C. Morris A. J. (2018). Cardiac-specific inactivation of LPP3 in mice leads to myocardial dysfunction and heart failure. *Redox Biol.* 14 261–271. 10.1016/j.redox.2017.09.015 28982073 PMC5635346

[B7] Chang C. L. Liao J. J. Huang W. P. Lee H. (2007). Lysophosphatidic acid inhibits serum deprivation-induced autophagy in human prostate cancer PC-3 cells. *Autophagy* 3 268–270. 17329959 10.4161/auto.3909

[B8] Chen H. Liu S. Liu X. Yang J. Wang F. Cong X. (2017). Lysophosphatidic acid pretreatment attenuates myocardial ischemia/reperfusion injury in the immature hearts of rats. *Front. Physiol.* 8:153. 10.3389/fphys.2017.00153 28377726 PMC5359218

[B9] Chen J. Chen Y. Zhu W. Han Y. Han B. Xu R. (2008). Specific LPA receptor subtype mediation of LPA-induced hypertrophy of cardiac myocytes and involvement of Akt and NFkappaB signal pathways. *J. Cell. Biochem.* 103 1718–1731. 10.1002/jcb.21564 17891781

[B10] Chen X. Yang X. Y. Wang N. D. Ding C. Yang Y. J. You Z. J. (2003). Serum lysophosphatidic acid concentrations measured by dot immunogold filtration assay in patients with acute myocardial infarction. *Scand. J. Clin. Lab. Invest.* 63 497–503. 14743959 10.1080/00365510310003265

[B11] Chen-Scarabelli C. Agrawal P. R. Saravolatz L. Abuniat C. Scarabelli G. Stephanou A. (2014). The role and modulation of autophagy in experimental models of myocardial ischemia-reperfusion injury. *J. Geriatr. Cardiol.* 11 338–348. 10.11909/j.issn.1671-5411.2014.01.009 25593583 PMC4294150

[B12] Contos J. J. Chun J. (2000). Genomic characterization of the lysophosphatidic acid receptor gene, lp(A2)/Edg4, and identification of a frameshift mutation in a previously characterized cDNA. *Genomics* 64 155–169. 10.1006/geno.2000.6122 10729222

[B13] Contos J. J. Chun J. (2001). The mouse lp(A3)/Edg7 lysophosphatidic acid receptor gene: genomic structure, chromosomal localization, and expression pattern. *Gene* 267 243–253. 11313151 10.1016/s0378-1119(01)00410-3

[B14] Cremers B. Flesch M. Kostenis E. Maack C. Niedernberg A. Stoff A. (2003). Modulation of myocardial contractility by lysophosphatidic acid (LPA). *J. Mol. Cell. Cardiol.* 35 71–80. 12623301 10.1016/s0022-2828(02)00279-1

[B15] Dai D. F. Johnson S. C. Villarin J. J. Chin M. T. Nieves-Cintron M. Chen T. (2011). Mitochondrial oxidative stress mediates angiotensin II-induced cardiac hypertrophy and Galphaq overexpression-induced heart failure. *Circ. Res.* 108 837–846. 10.1161/circresaha.110.232306 21311045 PMC3785241

[B16] Delbridge L. M. D. Mellor K. M. Taylor D. J. Gottlieb R. A. (2017). Myocardial stress and autophagy: mechanisms and potential therapies. *Nat. Rev. Cardiol.* 14 412–425. 10.1038/nrcardio.2017.35 28361977 PMC6245608

[B17] DeMartino G. N. (2018). Thematic minireview series - autophagy. *J. Biol. Chem.* 293 5384–5385. 10.1074/jbc.TM118.002429 29467224 PMC5900753

[B18] Dong L. Yuan Y. Opansky C. Chen Y. Aguilera-Barrantes I. Wu S. (2017). Diet-induced obesity links to ER positive breast cancer progression via LPA/PKD-1-CD36 signaling-mediated microvascular remodeling. *Oncotarget* 8 22550–22562. 10.18632/oncotarget.15123 28186980 PMC5410244

[B19] Fan G. P. Wang W. Zhao H. Cai L. Zhang P. D. Yang Z. H. (2015). Pharmacological inhibition of focal adhesion kinase attenuates cardiac fibrosis in mice cardiac fibroblast and post-myocardial-infarction models. *Cell. Physiol. Biochem.* 37 515–526. 10.1159/000430373 26330161

[B20] Fielitz J. Kim M. S. Shelton J. M. Qi X. Hill J. A. Richardson J. A. (2008). Requirement of protein kinase D1 for pathological cardiac remodeling. *Proc. Natl. Acad. Sci. U.S.A.* 105 3059–3063. 10.1073/pnas.0712265105 18287012 PMC2268584

[B21] Gu C. Wang F. Zhao Z. Wang H. Cong X. Chen X. (2017). Lysophosphatidic acid is associated with atherosclerotic plaque instability by regulating NF-kappaB dependent matrix metalloproteinase-9 expression via LPA2 in macrophages. *Front. Physiol.* 8:266. 10.3389/fphys.2017.00266 28496416 PMC5406459

[B22] Hecht J. H. Weiner J. A. Post S. R. Chun J. (1996). Ventricular zone gene-1 (vzg-1) encodes a lysophosphatidic acid receptor expressed in neurogenic regions of the developing cerebral cortex. *J. Cell Biol.* 135 1071–1083. 8922387 10.1083/jcb.135.4.1071 PMC2133395

[B23] Herrero-Martin G. Hoyer-Hansen M. Garcia-Garcia C. Fumarola C. Farkas T. Lopez-Rivas A. (2009). TAK1 activates AMPK-dependent cytoprotective autophagy in TRAIL-treated epithelial cells. *EMBO J.* 28 677–685. 10.1038/emboj.2009.8 19197243 PMC2666037

[B24] Hilal-Dandan R. Means C. K. Gustafsson A. B. Morissette M. R. Adams J. W. Brunton L. L. (2004). Lysophosphatidic acid induces hypertrophy of neonatal cardiac myocytes via activation of Gi and Rho. *J. Mol. Cell. Cardiol.* 36 481–493. 10.1016/j.yjmcc.2003.12.010 15081308

[B25] Kam Y. Exton J. H. (2004). Role of phospholipase D1 in the regulation of mTOR activity by lysophosphatidic acid. *FASEB J.* 18 311–319. 10.1096/fj.03-0731com 14769825

[B26] Kanamori H. Takemura G. Goto K. Maruyama R. Tsujimoto A. Ogino A. (2011). The role of autophagy emerging in postinfarction cardiac remodelling. *Cardiovasc. Res.* 91 330–339. 10.1093/cvr/cvr073 21406597

[B27] Karliner J. S. Honbo N. Summers K. Gray M. O. Goetzl E. J. (2001). The lysophospholipids sphingosine-1-phosphate and lysophosphatidic acid enhance survival during hypoxia in neonatal rat cardiac myocytes. *J. Mol. Cell. Cardiol.* 33 1713–1717. 10.1006/jmcc.2001.1429 11549349

[B28] Kim J. Kundu M. Viollet B. Guan K.-L. (2011). AMPK and mTOR regulate autophagy through direct phosphorylation of Ulk1. *Nat. Cell Biol.* 13 132–141. 10.1038/ncb2152 21258367 PMC3987946

[B29] Lee H. J. Ryu J. M. Jung Y. H. Lee K. H. Kim D. I. Han H. J. (2016). Glycerol-3-phosphate acyltransferase-1 upregulation by O-GlcNAcylation of Sp1 protects against hypoxia-induced mouse embryonic stem cell apoptosis via mTOR activation. *Cell Death Dis.* 7:e2158. 10.1038/cddis.2015.410 27010859 PMC4823928

[B30] Maeda H. Nagai H. Takemura G. Shintani-Ishida K. Komatsu M. Ogura S. (2013). Intermittent-hypoxia induced autophagy attenuates contractile dysfunction and myocardial injury in rat heart. *Biochim. Biophys. Acta* 1832 1159–1166. 10.1016/j.bbadis.2013.02.014 23499993

[B31] Mathew J. Sleight P. Lonn E. Johnstone D. Pogue J. Yi Q. (2001). Reduction of cardiovascular risk by regression of electrocardiographic markers of left ventricular hypertrophy by the angiotensin-converting enzyme inhibitor ramipril. *Circulation* 104 1615–1621. 11581138 10.1161/hc3901.096700

[B32] Matsui Y. Takagi H. Qu X. Abdellatif M. Sakoda H. Asano T. (2007). Distinct roles of autophagy in the heart during ischemia and reperfusion: roles of AMP-activated protein kinase and Beclin 1 in mediating autophagy. *Circ. Res.* 100 914–922. 10.1161/01.res.0000261924.76669.36 17332429

[B33] Mills G. B. Moolenaar W. H. (2003). The emerging role of lysophosphatidic acid in cancer. *Nat. Rev. Cancer* 3 582–591. 10.1038/nrc1143 12894246

[B34] Moolenaar W. H. Perrakis A. (2011). Insights into autotaxin: how to produce and present a lipid mediator. *Nat. Rev. Mol. Cell Biol.* 12 674–679. 10.1038/nrm3188 21915140

[B35] Nakai A. Yamaguchi O. Takeda T. Higuchi Y. Hikoso S. Taniike M. (2007). The role of autophagy in cardiomyocytes in the basal state and in response to hemodynamic stress. *Nat. Med.* 13 619–624. 10.1038/nm1574 17450150

[B36] Nsaibia M. J. Boulanger M. C. Bouchareb R. Mkannez G. Le Quang K. Hadji F. (2017). OxLDL-derived lysophosphatidic acid promotes the progression of aortic valve stenosis through a LPAR1-RhoA-NF-kappaB pathway. *Cardiovasc. Res.* 113 1351–1363. 10.1093/cvr/cvx089 28472283 PMC5852522

[B37] Ren B. Best B. Ramakrishnan D. P. Walcott B. P. Storz P. Silverstein R. L. (2016). LPA/PKD-1-FoxO1 Signaling Axis Mediates Endothelial Cell CD36 Transcriptional Repression and Proangiogenic and Proarteriogenic Reprogramming. *Arterioscler. Thromb. Vasc. Biol.* 36 1197–1208. 10.1161/atvbaha.116.307421 27013613 PMC4882231

[B38] Ren B. Hale J. Srikanthan S. Silverstein R. L. (2011). Lysophosphatidic acid suppresses endothelial cell CD36 expression and promotes angiogenesis via a PKD-1-dependent signaling pathway. *Blood* 117 6036–6045. 10.1182/blood-2010-12-326017 21441463 PMC3112047

[B39] Shen X. Zou J. Li F. Zhang T. Guo T. (2018). Lysophosphatidic acid enhances neointimal hyperplasia following vascular injury through modulating proliferation, autophagy, inflammation and oxidative stress. *Mol. Med. Rep.* 18 87–96. 10.3892/mmr.2018.8937 29749484 PMC6059717

[B40] Smyth S. S. Mueller P. Yang F. Brandon J. A. Morris A. J. (2014). Arguing the case for the autotaxin-lysophosphatidic acid-lipid phosphate phosphatase 3-signaling nexus in the development and complications of atherosclerosis. *Arterioscler. Thromb. Vasc. Biol.* 34 479–486. 10.1161/atvbaha.113.302737 24482375 PMC3944085

[B41] Tanaka Y. Guhde G. Suter A. Eskelinen E. L. Hartmann D. Lullmann-Rauch R. (2000). Accumulation of autophagic vacuoles and cardiomyopathy in LAMP-2-deficient mice. *Nature* 406 902–906. 10.1038/35022595 10972293

[B42] Taniguchi R. Inoue A. Sayama M. Uwamizu A. Yamashita K. Hirata K. (2017). Structural insights into ligand recognition by the lysophosphatidic acid receptor LPA6. *Nature* 548 356–360. 10.1038/nature23448 28792932

[B43] Teo S. T. Yung Y. C. Herr D. R. Chun J. (2009). Lysophosphatidic acid in vascular development and disease. *IUBMB Life* 61 791–799. 10.1002/iub.220 19621353 PMC4307796

[B44] van Meeteren L. A. Ruurs P. Stortelers C. Bouwman P. van Rooijen M. A. Pradere J. P. (2006). Autotaxin, a secreted lysophospholipase D, is essential for blood vessel formation during development. *Mol. Cell. Biol.* 26 5015–5022. 10.1128/mcb.02419-05 16782887 PMC1489177

[B45] Wei Y. Zhao L. Q. Qi B. Z. Xiao X. He L. Zhou G. Q. (2012). Lysophosphatidic acid increases the electrophysiological instability of adult rabbit ventricular myocardium by augmenting L-type calcium current. *PLoS One* 7:e45862. 10.1371/journal.pone.0045862 23029283 PMC3448719

[B46] Wu X. He L. Chen F. He X. Cai Y. Zhang G. (2014). Impaired autophagy contributes to adverse cardiac remodeling in acute myocardial infarction. *PLoS One* 9:e112891. 10.1371/journal.pone.0112891 25409294 PMC4237367

[B47] Xu Y. J. Rathi S. S. Zhang M. Bhugra P. Dhalla N. S. (2002). Mechanism of the positive inotropic effect of lysophosphatidic acid in rat heart. *J. Cardiovasc. Pharmacol. Ther.* 7 109–115. 10.1177/107424840200700207 12075399

[B48] Yang G. Wang N. Seto S. W. Chang D. Liang H. (2018). Hydroxysafflor yellow a protects brain microvascular endothelial cells against oxygen glucose deprivation/reoxygenation injury: involvement of inhibiting autophagy via class I PI3K/Akt/mTOR signaling pathway. *Brain Res. Bull.* 140 243–257. 10.1016/j.brainresbull.2018.05.011 29775658

[B49] Yang J. Nie Y. Wang F. Hou J. Cong X. Hu S. (2013). Reciprocal regulation of miR-23a and lysophosphatidic acid receptor signaling in cardiomyocyte hypertrophy. *Biochim. Biophys. Acta* 1831 1386–1394. 10.1016/j.bbalip.2013.05.005 23711961

[B50] Ye X. Hama K. Contos J. J. Anliker B. Inoue A. Skinner M. K. (2005). LPA3-mediated lysophosphatidic acid signalling in embryo implantation and spacing. *Nature* 435 104–108. 10.1038/nature03505 15875025 PMC1369590

[B51] Yoshida K. Nishida W. Hayashi K. Ohkawa Y. Ogawa A. Aoki J. (2003). Vascular remodeling induced by naturally occurring unsaturated lysophosphatidic acid in vivo. *Circulation* 108 1746–1752. 10.1161/01.cir.0000089374.35455.f3 14504178

[B52] Yu F. X. Zhao B. Panupinthu N. Jewell J. L. Lian I. Wang L. H. (2012). Regulation of the Hippo-YAP pathway by G-protein-coupled receptor signaling. *Cell* 150 780–791. 10.1016/j.cell.2012.06.037 22863277 PMC3433174

[B53] Yukiura H. Kano K. Kise R. Inoue A. Aoki J. (2015). Autotaxin overexpression causes embryonic lethality and vascular defects. *PLoS One* 10:e0126734. 10.1371/journal.pone.0126734 25992708 PMC4438000

[B54] Yung Y. C. Stoddard N. C. Chun J. (2014). LPA receptor signaling: pharmacology, physiology, and pathophysiology. *J. Lipid Res.* 55 1192–1214. 10.1194/jlr.R046458 24643338 PMC4076099

[B55] Zhang D. Zhang Y. Zhao C. Zhang W. Shao G. Zhang H. (2016). Effect of lysophosphatidic acid on the immune inflammatory response and the connexin 43 protein in myocardial infarction. *Exp. Ther. Med.* 11 1617–1624. 10.3892/etm.2016.3132 27168781 PMC4840587

[B56] Zhang H. Bialkowska A. Rusovici R. Chanchevalap S. Shim H. Katz J. P. (2007). Lysophosphatidic acid facilitates proliferation of colon cancer cells via induction of Kruppel-like factor 5. *J. Biol. Chem.* 282 15541–15549. 10.1074/jbc.M700702200 17430902 PMC2000347

[B57] Zhou Z. W. Li X. X. He Z. X. Pan S. T. Yang Y. Zhang X. (2015). Induction of apoptosis and autophagy via sirtuin1- and PI3K/Akt/mTOR-mediated pathways by plumbagin in human prostate cancer cells. *Drug Des. Devel. Ther.* 9 1511–1554. 10.2147/dddt.s75976 25834399 PMC4366042

